# Health literacy, consciousness, and locus of control in relation to vaccine hesitancy and refusal: Alexandros Heraclides

**DOI:** 10.1093/eurpub/ckac129.640

**Published:** 2022-10-25

**Authors:** K Kouvari, A Hadjikou, I Heraclidou, A Heraclides

**Affiliations:** 1 Health Sciences, European University Cyprus, Nicosia, Cyprus; 2 Psychology, University of Cyprus, Nicosia, Cyprus

## Abstract

**Background:**

About 35% of adult Europeans remain not fully vaccinated against COVID-19. Health literacy (HL), health consciousness (HC), and health locus of control (HLC) have been linked to different health behaviors, yet their role in COVID-19 vaccination uptake remains unclear. Here, we report preliminary findings from a cross-sectional survey conducted in Greece and Cyprus, aiming to elucidate the aforementioned associations.

**Methods:**

Participant recruitment for the current analysis took place from January to June 2022, following proportional quota sampling. The current sample comprises 190 participants, with full information on COVID-19 vaccine hesitancy (composite scale outcome) and refusal (binary outcome), as well as HL (European Health Literacy Survey Questionnaire-Q16), HC (Health Consciousness Scale-G), and HLC (Multidimensional Health Locus of Control Form B). Linear and logistic regression analyses were used to determine associations between the aforementioned factors, using the standardized versions of the independent variables.

**Results:**

After adjusting for sociodemographic factors and other predictors of vaccination status, HL (β, 95% CI: -2.56, -3.76; -1.36), ‘internal’ HLC (-2.76, -3.79; -1.73) and ‘health professionals’ HLC (-2.64, -3.66; -1.63) were strongly and negatively associated with vaccine hesitancy, while HC showed a weaker association in the same direction (-1.46, -2.58; -0.35). In contrast, ‘chance’ HLC was strongly and positively associated with vaccine hesitancy (4.40, 3.37; 5.43). Similar results were detected when vaccine refusal was used as a binary outcome.

**Conclusions:**

Health literacy and locus of control and to a lesser extent health consciousness, are independent predictors of COVID-19 vaccine hesitancy and refusal. Increasing vaccination uptake via programs aiming at enhancing health literacy and shifting health locus of control, can have a significant impact on COVID-19 pandemic management internationally.

**Key messages:**

• Health literacy, locus of control and to a lesser extent consciousness, are novel predictors of vaccine hesitancy and refusal in two Eastern Mediterranean European populations.

• Increasing health literacy and shifting health locus of control could render programs aiming to increase COVID-19 vaccine uptake more targeted and effective, internationally.

